# Design and Characterization of Auxotrophy-Based Amino Acid Biosensors

**DOI:** 10.1371/journal.pone.0041349

**Published:** 2012-07-19

**Authors:** Felix Bertels, Holger Merker, Christian Kost

**Affiliations:** Research Group Experimental Ecology and Evolution, Max Planck Institute for Chemical Ecology, Jena, Germany; University of Groningen, The Netherlands

## Abstract

Efficient and inexpensive methods are required for the high-throughput quantification of amino acids in physiological fluids or microbial cell cultures. Here we develop an array of *Escherichia coli* biosensors to sensitively quantify eleven different amino acids. By using online databases, genes involved in amino acid biosynthesis were identified that – upon deletion – should render the corresponding mutant auxotrophic for one particular amino acid. This rational design strategy suggested genes involved in the biosynthesis of arginine, histidine, isoleucine, leucine, lysine, methionine, phenylalanine, proline, threonine, tryptophan, and tyrosine as potential genetic targets. A detailed phenotypic characterization of the corresponding single-gene deletion mutants indeed confirmed that these strains could neither grow on a minimal medium lacking amino acids nor transform any other proteinogenic amino acid into the focal one. Site-specific integration of the *egfp* gene into the chromosome of each biosensor decreased the detection limit of the GFP-labeled cells by 30% relative to turbidometric measurements. Finally, using the biosensors to determine the amino acid concentration in the supernatants of two amino acid overproducing *E. coli* strains (i.e. *ΔhisL and ΔtdcC*) both turbidometrically and via GFP fluorescence emission and comparing the results to conventional HPLC measurements confirmed the utility of the developed biosensor system. Taken together, our study provides not only a genotypically and phenotypically well-characterized set of publicly available amino acid biosensors, but also demonstrates the feasibility of the rational design strategy used.

## Introduction

The rapid and exact determination of amino acid concentrations is of fundamental importance for a wide range of applications including biological, medical, or food technological analyses. For example, abnormal levels of amino acids in human blood are diagnostic for amino acid disorder diseases [1], in animal nutrition concentrations of essential amino acids have a vital influence on animal weight gain [2], [3], and in fermentation processes it is important to continuously monitor the consumption or production of certain amino acids [4], [5]. Especially so-called combinatorial approaches that are frequently used in metabolic engineering of microbial strains to overproduce certain amino acids of interest rely on the rapid screening of large mutant libraries [6].

One way to quantify the amount of free amino acids in complex matrices such as blood or bacterial culture media is to apply methods of analytical chemistry. Here, complex mixtures of compounds are separated via liquid- or gas chromatography and subsequently the amount of amino acids is quantified using fluorescence-, UV-detection or mass spectrometry [7], [8], [9], [10]. However, drawbacks of these approaches are not only that the analyses are often very time-consuming, but also that these techniques provide no information on the bioavailability and bioaccessability of the respective amino acids [11]. Moreover, depending on the analytical methodology used, technical difficulties like matrix interference, a low sensitivity, elaborate sample preparation procedures, multiplicity of peaks formed, or a bias of the derivatising agent used against certain amino acids can constrain the applicability of the technique as well as the validity of the results (see [12], [13] and references therein).

These problems can be overcome by using amino acid biosensors, which use either enzymes (i.e. L-amino acid oxidase, L-AAO) or whole cells as a biological recognition element [14]. The enzymes are coupled to an amperometric sensor, which measures the enzymatic activity, e.g. via the consumption of oxygen [15] or the production of hydrogen peroxide [16], or both [17]. These biosensors allow for example continuous monitoring of the amino acid concentration during fermentation processes.

In the whole cell approach, either naturally occurring (e.g. *Lactobacillus arabinosus*, *Leuconostoc mesenteroides*) or genetically modified amino acid auxotrophic bacteria (e.g. *E. coli*) are used [18], [19], [20]. By comparing the growth of these bacteria in an environment that contains the amino acid in question with their growth under known amino acid concentrations, the amino acid concentration in the growth medium can be determined. The growth of such auxotrophs has been determined in different ways, including the measurement of medium acidification [21], the culture's optical density (OD) [18], or by quantifying previously integrated phenotypic markers such as ß-galactosidase activity [22], fluorescence [3], or luminescence [23].

The ideal biosensor used for the quantification of amino acids should be i) relatively fast growing, ii) able to also grow in minimal media, iii) deficient in converting other amino acids into the focal one, iv) insensitive to in its growth phenotype to other constituents of the medium than amino acids, and v) publicly available. Among the bacterial strains that have been used for this purpose, *E. coli* fulfills most of the above-mentioned criteria: it has simple media requirements, grows fast, and the ease with which amino acid auxotrophs can be generated makes it a fast, simple and inexpensive tool for the determination of amino acid levels in a high-throughput manner [18]. Most of the existent biosensors, however, were created by mutagen treatment or transposon mutagenesis [24], [25], which both randomly introduce mutations into the bacterial genome. As a consequence, in many cases the exact chromosomal location of the auxotrophy-causing mutation remains unknown. Moreover, virtually none of the amino acid biosensors available to date have been subjected to a more detailed phenotypic characterization to verify as to which extend the target strain is able to chemically convert other amino acids into the focal one, thereby overcoming the auxotrophy.

**Table 1:** Bacterial strains and plasmids used in this study.

Bacterial strains and plasmids	Relevant characteristics	Reference
***Escherichia coli***		
BW25113	Wild type	[26]
JW3932	Arginine auxotroph, *ΔargH*, (argininosuccinate lyase)	[26]
JW2002	Histidine auxotroph, *ΔhisD*, (histidinal dehydrogenase)	[26]
JW3745	Isoleucine auxotroph, *ΔilvA*, (threonine deaminase)	[26]
JW5807	Leucine auxotroph, *ΔleuB*, (3-isopropylmalate dehydrogenase)	[26]
JW2806	Lysine auxotroph, *ΔlysA*, (diaminopimelate decarboxylase)	[26]
JW3910	Methionine auxotroph, *ΔmetB (*O-succinylhomoserine lyase)	[26]
JW2580	Phenylalanine auxotroph, *ΔpheA* (chorismate mutase/ prephenate dehydratase)	[26]
JW0377	Proline auxotroph, *ΔproC*, (pyrroline-5-carboxylate reductase)	[26]
JW0003	Threonine auxotroph, *ΔthrC*, (threonine synthase)	[26]
JW1254	Tryptophan auxotroph, *ΔtrpC*, (indole-3-glycerol phosphate synthase)	[26]
JW2581	Tyrosine auxotroph, *ΔtyrA*, (chorismate mutase/ prephenate dehydrogenase)	[26]
JW3087	Tryptophan overproducer, *ΔtdcC*, (threonine STP transporter)	[26]
JW2000	Histidin overproducer, JW2000, *ΔhisL*, (his operon leader peptide)	[26]
**Plasmids**		
pGRG36	Contains Tn7 transposase gene *tnsABCD*	[33]
pJBA24-*egfp*	Contains expression cassette: P*_lacZ_*-RBSII-*egfp*-t_o_-t_1_-*egfp*	[31]
pGRG36-P*_lacZ_*-*egfp*	*tnsABCD,* P*_lacZ_*-RBSII-*egfp*-t_o_-t_1_	This study

To fill this gap, we developed an array of different, genetically well-characterized amino acid biosensors and subjected them to a detailed phenotypic analysis. Taking advantage of the resources that are publicly available for *E. coli*, we first performed an *in silico* analysis using online databases to identify potential genetic targets, which – upon deletion – would generate a strain auxotrophic for a certain amino acid. In a second step, the identified candidates were tested *in vivo* by measuring the growth of the respective single-gene deletion mutants (derived from the ‘Keio collection’ [26]) under various environmental conditions to determine the specificity of the corresponding knockout. Thirdly, the gene coding for the enhanced green fluorescent protein (EGFP, [27]) was introduced into the chromosome of the obtained biosensors to verify, whether the emitted fluorescent signal decreases the detection limit of the labeled cells over spectrophotometrical measurements of the OD [3]. Finally, to scrutinize the applicability of the developed biosensors, the amino acid production profiles of two *E. coli* strains overproducing tryptophan and histidine were quantified and the results compared to RP-HPLC measurements.

**Figure 1:**
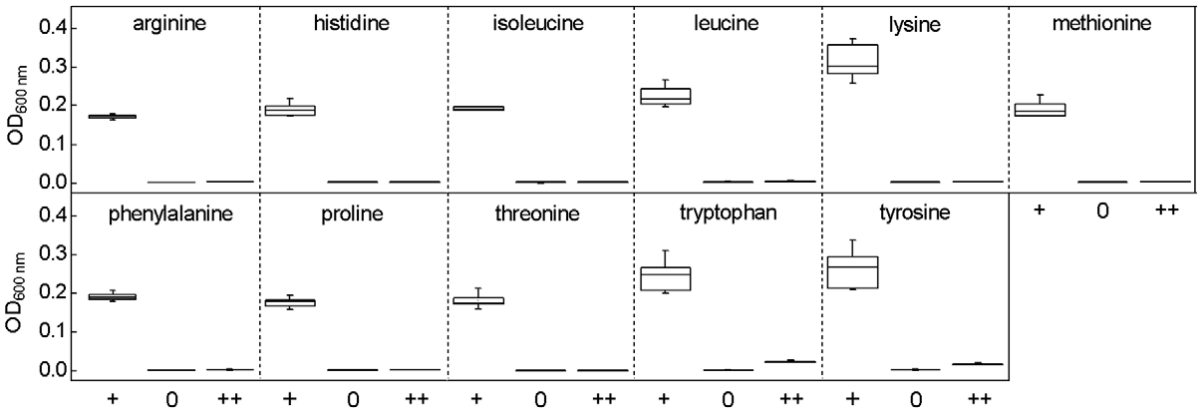
Confirmation of the biosensors' auxotrophy. The eleven biosensors were grown in minimal medium, which was supplemented with 3 mM of the required focal amino acid (**+**), devoid of any amino acid supplementation (**0**), or supplemented with 3 mM of each of the 19 other, proteinogenic amino acids (**++**). Growth of eight replicates was determined turbidometrically (OD_600 nm_) after 18 h (histidine, arginine, tryptophan, isoleucine, methionine, phenylalanine, tyrosine, proline, and lysine) or 24 h (leucine and threonine) of cultivation. Boxplots: median (horizontal lines in boxes), interquartile range (boxes), 1.5-fold interquartile range (whiskers).

## Materials and Methods

### *In silico* analysis

For the analysis of the amino acid biosynthetic pathways and the identification of candidate genes, which upon deletion would generate a strain auxotrophic for a certain amino acid, the KEGG-pathway [28] and the EcoCyc database [29] were used. First, all amino that *E. coli* can generate by using more than one biosynthetic pathway acids were excluded from further analysis. Within the remaining amino acid biosynthetic pathways it was carefully examined whether or not other amino acids could be biochemically transformed into the focal amino acid. If this was the case, the corresponding amino acid was excluded from further analysis. Finally, genes, whose products catalyze the terminal step of the corresponding biosynthetic pathway were selected as candidates for further analysis.

**Figure 2:**
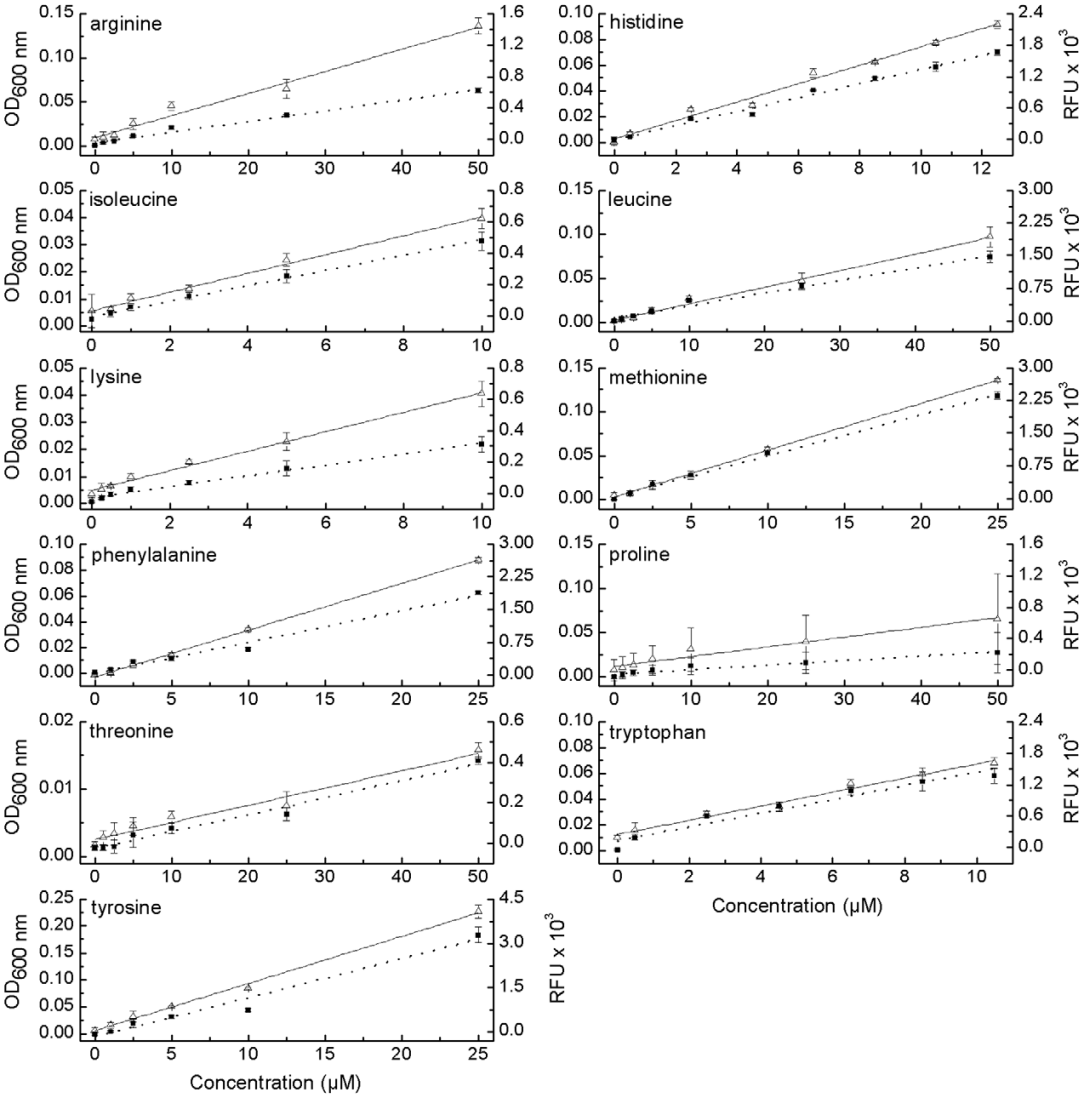
Amino acid-dependent growth of the eleven GFP-labeled biosensors. Each biosensor was cultivated in minimal medium, to which increasing concentrations of the required focal amino acid have been added. Cell growth was measured either turbidometrically (OD_600 nm_, ▪) or as relative fluorescence units (RFU, Δ) after 18 h (histidine, arginine, tryptophan, isoleucine, methionine, phenylalanine, tyrosine, proline and lysine) or 24 h (leucine and threonine) of cultivation. Means (±95% confidence interval) of four replicates are given.

**Table 2:** Coefficients of determination (R^2^) of calibration curves generated from amino acid-dependent growth of the different amino acid biosensors tested.

Biosensor	Deleted gene	OD_600 nm_ (R^2^)	RFU (R^2^)
Arginine	*ΔargH*	0.987	0.985
Histidine	*ΔhisD*	0.991	0.988
Isoleucine	*ΔilvA*	0.996	0.994
Leucine	*ΔleuB*	0.987	0.992
Lysine	*ΔlysA*	0.985	0.994
Methionine	*ΔmetB*	0.996	0.999
Phenylalanine	*ΔpheA*	0.984	0.999
Proline	*ΔproC*	0.943	0.960
Threonine	*ΔthrC*	0.981	0.969
Tryptophan	*ΔtrpC*	0.952	0.986
Tyrosine	*ΔtyrA*	0.973	0.997

Biosensor growth was determined either turbidometrically (OD_600 nm_) or by measuring GFP fluorescence emission (RFU).

### Bacterial strains and pre-culture conditions

The computationally identified putative amino acid auxotrophs as well as two amino acid overproducing strains were obtained from the Coli Genetic Stock Center (CGSC), where a set of *E. coli* – single-gene deletion mutants, the Keio collection, is deposited ([26], Table 1). For all experiments, strains were pre-cultured as follows: strains were streaked out from a −80°C glycerol stock on Lysogeny Broth (LB) plates and incubated over night at 37°C. The next day, single colonies were picked and used to inoculate LB liquid medium (2 ml), which were incubated at 37°C and 220 rpm for 12 h. A volume of 200 µl was centrifuged at 5,000 *g* for 3 min to harvest the cells and washed twice (200 µl) with MMAB minimal medium [30] with fructose (5 g l^−1^) and diluted to an OD_600 nm_ of 0.1. This diluted culture was used to inoculate the main culture 1∶100, which consisted of MMAB medium supplemented with fructose (5 g l^−1^).

**Figure 3:**
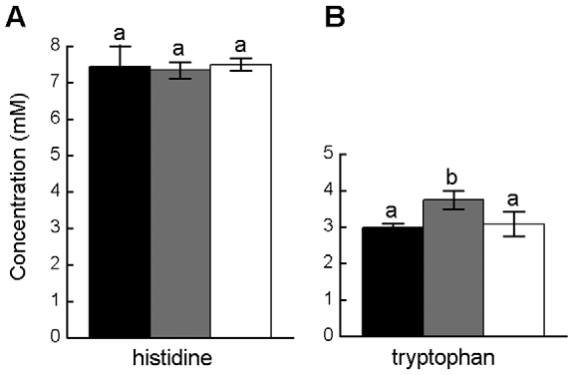
Quantification of amino acid concentrations in the culture supernatant of two mutant *E. coli* strains. Mean (±95% confidence interval) amount of (A) histidine produced by *E. coli ΔhisL*, and (B) tryptophan produced by *E. coli ΔtrpcC* as determined by conventional HPLC measurements (black bar) or the growth of the respective biosensors, which was quantified turbidometrically (OD_600 nm_, grey bar) or GFP fluorescence emission (white bar). Different letters indicate significant differences (S-N-K test: P<0.05, n = 4).

### Construction of EGFP- labeled amino acid biosensors

The construction of pGRG-P*_lacZ_*-*egfp* was based on the plasmid pJBA24-*egfp* [31] where *egfp* is integrated between an artificial ribosomal binding site II (RBSII) and transcriptional terminators t_0_ and t_1_. An artificial *lacZ* promotor called P_A1/04/03_ [32] upstream of RBSII controls the expression of EGFP. From this plasmid the expression-cassette P*_lacZ_*-RBSII-*egfp*-t_0_-t_1_ was excised by the restriction enzyme NotI and ligated into the multiple cloning site of pGRG36 (Addgene plasmid 21322) to generate pGRG-P*_lacZ_*-*egfp* (Table 1). pGRG36 [33] harbors genes which encode for Tn7 transposases and a multiple cloning site, which is flanked by the terminal repeats *Tn7-L* and *Tn7-R*. pGRG-P*_lacZ_*-*egfp* was cloned into the identified auxotrophic mutants as well as the *E. coli* wild type BW25113, which were treated according to the transgene insertion protocol of McKenzie and Craig (2006) to label them chromosomally with the expression cassette. The success of this step was confirmed by a diagnostic PCR on the expected region with the primers (5′-GATGCTGGTGGCGAAGCTGT-3′) and (5′GATGACGGTTTGTCACATGGA-3′).

**Table 3:** Comparison of the single-gene deletions that were identified in this study to result in specific amino acid auxotrophies of *E. coli* with published information on predicted and empirically tested amino acid auxotrophies.

	This study		Predicted		Tested	
Amino acid	Deleted gene	Effect	Deleted gene	Effect	Deleted gene	Effect
Arginine	*argH*	C	*argH*	C^1^	*argH*	B^2^
Histidine	*hisD*	C	*hisB*	C^1^	*hisI*	B^2^’
Isoleucine	*ilvA*	C	*ilvA*	C^1^	*ilvA*	B^2^
Leucine	*leuB*	C	*leuB*	C^1^	*leuB*	B^2^’
Lysine	*lysA*	C	*lysA*	C^1^	*lysA*	B^2^, C^3^
Methionine	*metB*	C	*metB*	C^1^	*metB*	B^2^’
Phenylalanine	*pheA*	C	*pheA*	C^1^	*pheA*	B^2^’
Proline	*proC*	C	*proB*	C^1^	*proC*	B^2^
Threonine	*thrC*	C	*thrC*	C^1^	*thrC*	B^2^
Tryptophan	*trpC*	B	*trpC*	C^1^	*trpC*	B^2^’
Tyrosine	*tyrA*	B	*tyrA*	C^1^	*tyrA*	B^2^

1  =  Tepper & Shlomi (2011), 2  =  Baba (2006), 3  =  Li & Ricke (2003), ‘  =  strain shows weak growth.

The effect of the corresponding deletion is indicated as: **A** strongly reduced growth of mutant on minimal medium, **B** mutant does not grow on minimal medium, **C** mutant cannot transform any other amino acid into the focal one, and **ns** degree of auxotrophy not specified.

### Verification of auxotrophy

To verify the amino acid auxotrophy of the deletion mutants *in vivo*, eight single colonies of each auxotroph (Table 1) were pre-cultured as described above. Each clone was used to inoculate three different media: i) MMAB medium supplemented with 3 mM of the focal amino acid (i.e. positive control), ii) MMAB medium without amino acids (i.e. negative control), and iii) MMAB medium supplemented with 3 mM of all other proteinogenic amino acids (i.e. control for biochemical transformation into focal amino acid). Cultures were incubated in 96 deep-well plates (1 ml per well) at 37°C and 220 rpm. After 12 h, 18 h, and 24 h the OD_600 nm_ was determined.

### Cultivation and supernatant harvest of amino acid overproducers

Both amino acid overproducers (*ΔhisL* and *ΔtdcC*) were pre-cultured as described above and inoculated into Erlenmeyer flasks (200 ml) 1∶100 containing 40 ml MMAB medium with fructose (5 g l^−1^). The cultures were incubated at 30°C and 220 rpm for 34 h, reaching an OD_600 nm_ of 0.78 and 0.13 for the *ΔhisL* and the *ΔtdcC* mutant, respectively. Subsequently, the two cultures were sterile-filtered through a 0.22 µm syringe filter and the amino acid concentration determined by biosensors and HPLC-FD, respectively. For the biosensor measurements, the overproducers' supernatants were diluted using sterile MMAB medium with fructose such that the observed growth of the biosensors would fall within the linear range of their calibration curves.

### Quantification of amino acid concentrations using biosensors

The pre-cultured biosensors (one colony each) were used to inoculate MMAB medium (1 ml) that contained kanamycin (50 µg µl^−1^), isopropyl-β-D-thio-galactoside (IPTG, 1 mM), and either the respective amino acid concentration as standard or the supernatant derived from one of the two amino acid overproducers (*ΔhisL* and *ΔtdcC*). This one milliliter was composed of 0.6 ml of 1.67-times concentrated MMAB medium and 0.4 ml of supernatant, diluted supernatant, or water with a defined amino acid concentration ranging from 0.5 µM to 50 µM. Each amino acid concentration/ supernatant-biosensor combination was replicated four times. After incubation for 12 h, 18 h and 24 h at 37°C and 220 rpm, 100 µl of each culture were used to determine fluorescence emission and OD_600 nm_ with a microplate reader (Tecan Infinite F200, Tecan Group Ltd., Männedorf, Switzerland). For fluorescence measurements, the gain was set to 100, excitation and emission wavelength to 485 nm and 515 nm respectively. The OD_600 nm_ and the RFU values were plotted against the added amino acid concentrations to generate calibration curves. The line equations of these calibration curves were used to calculate the amino acid concentration in the bacterial supernatants.

### Amino acid quantification by reversed phase-high-performance liquid chromatography (RP-HPLC)

Amino acid analysis was performed on a chromolith performance RP-18e (100 mm x 4.6 mm, Merck KGaA, Darmstadt, Germany) column with o-phtaldialdehyd (OPA) pre-column derivatisation. Each sample was pre-mixed with potassium borat buffer (0.5 M, pH 11) to a final volume of 100 µl. Derivatisation was performed automatically by an autosampler that added 30 µl of a mixture of OPA (85 mM) and β-mercaptoethanol (130 mM) to the sample, incubated it for 2 min at room temperature and loaded 30 µl onto the column. The mobile phase consisted of a mixture of solvent A (sodium citrate buffer 0.02 M, pH 5.5) and solvent B (acetonitril (35%, v/v) and methanol (65%, v/v)). In the beginning, 15% of solution B was used, which rose to 45% in the course of 28 min. The flow rate was set to 1.5 ml min^−1^. After 28 min, the mobile phase was switched to 100% solvent B for 2 minutes at a flow rate of 2 ml min^−1^. After that the column was reconstituted for 1.5 min with 15% of solvent B at a flow rate of 2 ml min^−1^ before the next analysis cycle started. The amino acid-OPA derivatives were quantified using a fluorescence detector (Ex: 340 nm, Em: 445 nm). The calibration was done in the same way using an amino acid mix in concentrations ranging from 5 µM to 50 µM (Sigma Aldrich, Taufkirchen, Germany).

### Determination of the limits of fluorescence and OD detection

In order to compare the detection limits of measuring either culture turbidity (i.e. OD) or fluorescence emission, six *E. coli* BW25113 clones that have been chromosomally labeled with *egfp* were cultivated over night as mentioned above. The cultures were diluted seven times 1∶2 and OD_600 nm_ and GFP fluorescence was measured. To compare these two methods, we additionally determined the cell number by flow-cytometry (CyFlow space, Partec, Münster, Germany). For this, cells were stained by adding 10 µl of SYBR green (Eurogentec, Köln, Germany). Fluorescence and OD were plotted against the cell number and the detection limits were calculated as described by Eggins (2007).

### Statistical analysis

The *accuracy* of a measurement reflects its closeness to the real value, while the *precision* measures the closeness of repeated measurements to the same value [34]. Accordingly, the *accuracy* of the biosensor measurements was determined by comparing OD or fluorescence measurements of the amino acid overproducer's supernatant to the HPLC-derived values using an univariate ANOVA followed by a Student-Newman-Keuls (S-N-K) posthoc test. The precision of OD and fluorescence measurements was determined by calculating the residuals of the amino acid calibration curves [34] and comparing them between the two detection methods. For this, a Levene's test was applied with the ‘detection method’ as fixed and the ‘amino acid concentration’ as covariate. All statistical analyses were performed using SPSS 17.0 (SPSS Inc., Chicago, USA).

## Results

### *In silico* analysis

A step-wise procedure was followed to identify genes in amino acid biosynthetic pathways of *E. coli*, whose deletion should result in specific amino acid auxotrophies in the corresponding mutants. First, all biosynthetic pathways were excluded for which more than one gene would have to be deleted to engineer an amino acid autotrophy. This was the case for aspartate (2), glutamate (3) and alanine (3), which *E. coli* can generate using multiple biosynthetic pathways. The second criterion of exclusion was that *E. coli* should not be able to transform any other proteinogenic amino acid into the focal one. Of the remaining biosynthetic pathways, both glycine and serine fell into this category. Besides the unique biosynthetic pathway leading to these two amino acids, *E. coli* can also generate them using threonine and glycine as precursors, respectively. Moreover, the only biosynthetic step to yield glutamine and asparagine from the respective carboxylized precursors glutamate and aspartate can be catalyzed by more than one enzyme. Hence, two or more mutations would have been required to obtain a specific auxotroph for these two amino acids. Finally, the biosynthetic pathway leading to valine is partially overlapping with the one required for isoleucine production. Hence, deleting one of these genes should result in a double-auxotrophy for both amino acids. The last amino acid we excluded from further analysis was cysteine, because of the spontaneous formation of cystin under oxic conditions, which cannot be utilized by *E. coli* BW25113. After excluding these nine amino acids, the following amino acids were identified as potential targets for further analysis (predicted deletion in brackets): arginine (*ΔargH*), histidin (*ΔhisD*), isoleucine (*ΔilvA*), leucine (*ΔleuB*), lysine (*ΔlysA*), methionine (*ΔmetB*), phenylalanine *(ΔpheA*), proline (*ΔproC*), threonine (*ΔthrC*), tryptophan (*ΔtrpC*), and tyrosine (*ΔtyrA*). Here, the terminal biosynthetic steps were selected to interrupt each pathway by gene deletion and the corresponding mutants obtained from the *Coli* Genetic Stock Center (CGSC).

### Assay duration

To identify the optimal time span required for each assay, we first determined when the growth of the particular auxotrophs reached a maximum density in an environment, in which the required amino acid was not growth-limiting. This analysis suggested 18 h as the optimal time point for all tested mutants (Fig. S1). Only for the threonine- and leucine-auxotrophic mutants 24 h was chosen as the incubation time in subsequent experiments, because the precision of the calibration curves of these two mutants was greater for 24 h than for the 18 h time point. Despite the fact that the the population density of the tryptophan auxotroph was already declining after 12 h (Fig. S1) an assay duration of 18 h was chosen for this mutant as well, because the fluorescence signal emitted by the EGFP-labeled deletion mutant was strongest at this time point (Fig. S2).

### Verification of auxotrophy

Based on the *in silico* analysis, it was expected that the putative amino acid auxotrophs should neither be able to grow in minimal medium without amino acid supplementation, nor should they be able to transform other amino acids into the focal one. These predictions were tested by determining the population density of each identified deletion mutant in three growth environments at the previously determined time point. This analysis confirmed indeed that all eleven amino acid biosensors essentially required the focal amino acid for growth and could not transform any other proteinogenic amino acid into the focal one (Fig. 1). Exceptions to this were the *ΔtrpC* and *ΔtyrA* mutants that showed weak growth after 18 h of cultivation (Fig. 1).

### Amino acid-dependent growth of the biosensors

A calibration curve was generated by cultivating each identified biosensor in minimal medium, which has been supplemented with different concentrations of the corresponding amino acids. Incubating each biosensor for the previously determined time and subsequently determining its growth both turbidometrically and by measuring EGFP fluorescence emission indicated a linear relationship between the growth response of the different populations and the concentration of amino acids present in the growth environment (Fig. 2). The growth of the auxotrophs for histidine, tryptophan, isoleucine and lysine responded linearly to increasing amino acid concentrations in the range from 0.5 µM to 10 µM. For the methionine, phenylalanine, and tyrosine auxotrophs, this range was between 0.5 µM and 25 µM, and for the arginine, leucine, threonine, and proline biosensors between 1 µM and 50 µM (Fig. 2). All calibration curves showed a very strong linearity between both OD and fluorescence measurements and the target amino acid concentration as indicated by coefficients of determination (R^2^) that were generally ≥0.97 (Table 2). The only exceptions to this were the coefficients of determination for the calibration curves of proline (OD_600 nm_ and RFU), tryptophan (OD_600 nm_), and threonine (RFU), which showed R^2^ values ≥0.94, ≥0.95, and ≥0.96, respectively (Table 2).

### Evaluation of the amino acid biosensors

We hypothesized that measuring GFP fluorescence emission rather than optical density of a culture to quantify bacterial growth should significantly decrease the detection limit and hence increase the sensitivity of the bioassay. To test this, the population density of a serially-diluted culture of GFP-labelled *E. coli* WT cells was quantified by both detection methods and correlated to the absolute cell number of each culture as determined by flow-cytometry. This analysis indicated that the detection limit of GFP-fluorescence was 1.47±0.15×10^8^ cells ml^−1^ and of the OD_600 nm_ measurements 2.03±0.11×10^8^ cells ml^−1^. Hence, using fluorescence emission rather than culture turbidity to estimate the population density resulted in a significant improvement of the detection limit (paired t-test: P≤0.01) by almost 30%, which is in line with the abovementioned hypothesis.

The *precision* of both biosensor detection methods as estimated by comparing the residuals of the two calibration curves was not significantly different between the two methods for any of the eleven amino acids tested (Levene's test: P>0.05). The only exception to this was the threonine biosensor whose GFP fluorescence showed a significantly larger variance by 35% than the corresponding turbidometric measurements (Levene's test: P = 0.014).

The *accuracy* of the newly-developed biosensor system was verified by determining the concentration of amino acids in the culture supernatant of two amino acid overproducing strains of *E. coli* using the eleven biosensors and comparing the results to conventional HPLC measurements. For this, both OD_600 nm_ and RFU values were determined. In this way, 7.4 mM histidine was detected in the supernatant of the *ΔhisL* mutant and 3 mM tryptophan in the *ΔtdcC* supernatant (Fig. 3). None of the other biosensors detected any other amino acid in the two culture supernatants tested (data not shown). In case of the histidine-containing *ΔhisL* supernatant, the amino acid concentrations determined by HPLC and both biosensor detection methods were statistically indistinguishable (Fig. 3, S-N-K posthoc test: P>0.05). In contrast, when the tryptophan concentration of the *ΔtdcC* mutant's supernatant was analysed, only the fluorescence-based biosensor detection revealed the exact same amino acid concentration as the HPLC measurement (S-N-K posthoc test: P>0.05), whereas the turbidity measurements significantly overestimated the concentration of tryptophan in the analysed supernatant by 26% (Fig. 3, S-N-K posthoc test: P>0.05). Hence, the *accuracy* of the biosensor detection method applied depended on the individual biosensor used and – for the two amino acids analysed – was greater for the fluorescence-based biosensor detection.

## Discussion

The main goal of this work was to develop and test a system of auxotrophy-based biosensors that can be used to quantify the amount of amino acids in various solutions. Eleven genes involved in amino acid biosynthesis were identified *in silico* by applying a rational design strategy and the corresponding single gene deletion mutants obtained from the ‘Keio collection’ [26]. A subsequent phenotypic characterization of these mutants indicated indeed that the predicted deletions rendered the resulting strains incapable of growing in amino acid-free minimal medium and transforming any other proteinogenic amino acid into the focal one. The only exception to the latter were the tryptophan and tyrosin-auxotrophic mutants (*ΔtrpC and ΔtyrA*) that displayed weak growth. Strong linear relationships between the growth of the eleven biosensors and the concentration of the focal amino acids in the growth environment established the utility of the deletion mutants as amino acid biosensors. A comparison between turbidometric and GFP-fluorescence-based cell detection indicated that using GFP as a marker can increase the method's sensitivity. Besides two exceptions, the *precision* and *accuracy* were generally comparable between a fluorescence-based and a turbidometric determination of the biosensor's growth.

A variety of approaches have been used to develop whole-cell biosensors to rapidly detect and quantify certain chemicals in biological fluids. Among them, random mutagenesis approaches followed by systematic screening processes have been very successfully applied to generate specific biosensors [24], [25]. The drawback of this approach, however, is that the genetic basis underlying the engineered auxotrophy often remains obscure. This fact can hinder for example the construction of more complex biosensors that integrate more than one environmental signal [14]. To overcome this issue, prior knowledge can be used to rationally design the desired biosensors. In this study we have taken advantage of publicly available databases to identify suitable genetic targets. Interestingly, the candidate genes identified in this way closely matched predictions of a theoretical study (Table 3), in which knockouts giving rise to amino acid auxotrophies have been determined computationally by modeling a genome-scale metabolic network of *E. coli* [6]. The strategy employed by these authors combines constraint-based modeling with flux balance analysis to predict so-called ‘ultra-auxotrophs’. These are genotypes, whose growth depends exclusively on the absence/ presence of the focal metabolite and is independent of other metabolites in the growth environment. Mismatches between the target genes predicted in [6] and our study occurred within the biosynthetic pathways of proline, histidine, and arginine (Table 3), for which Tepper and Shlomi (2011) suggest to delete different genes within the same pathway.

Experimentally verifying the degree of auxotrophy in different growth environments confirmed the predicted growth phenotypes and suggested ultra-auxotrophies for almost all biosensors tested (Fig. 1). Moreover, the phenotypic effects observed were well in accord with previously published information (Table 3). Specifically, none of the auxotrophs tested showed considerable growth in a minimal medium lacking amino acids [26]. Also our finding that a deletion mutant for the diaminopimelate decarboxylase (*lysA*) gene was not able to transform any other proteinogenic amino acid into the focal one (Fig. 1) was supported by previous experimental evidence [26], [35].

The observation that the *trpC* deletion mutant showed some weak growth when all proteinogenic amino acids except tryptophan were supplied to the culture medium may be explained as follows: Under amino acid-rich growth conditions *ΔtrpC* cells may release indole into the environment [36], which is then taken up again (e.g. via the *mtr* transporter) and subsequently condensed by the tryptophan synthase (*trpB*) to the externally supplied serine to yield tryptophan [6]. The question why also the *ΔtyrA* mutant showed some weak growth under these conditions remains unclear and warrants future investigation. Nevertheless did the observed weak growth in amino acid containing environments not impair the applicability of both the *ΔtrpB* and the *ΔtyrA* mutant as biosensor for tryptophan and tyrosin, respectively (Fig. 3). Together, these results highlight the great accuracy, with which the *E. coli* metabolic network has been annotated that afforded the required predictive power to the rational design strategy used here. Due to the enormous speed with which metabolic networks are currently reconstructed [37], rational strategies to engineer desired phenotypes (e.g. [6], this study) will soon become applicable to other bacterial species as well. Furthermore, the good agreement between the theoretical predictions made by Tepper and Shlomi (2011) and the experimental verifications in our study, suggests that it would be worthwhile to similarly scrutinize also the other auxotrophs suggested in [6] for their potential use as biosensors. These include auxotrophic mutants for eight additional amino acids as well as sugars, nucleosides/ nucleotides, fats/ lipids, and other metabolites. Moreover, the spectrum of available *E. coli* biosensors might even be further increased by also considering gene up- or down-regulations as well as insertions of additional genes [6].

To improve the detection limits of the newly developed biosensors, all auxotrophs were chromosomally labeled with *egfp* by Tn*7* transposon mutagenesis. In this way, the protein levels per cell were more constant relative to integrating the marker into a plasmid that itself shows considerable variation in its copy number (FB, personal observation). Moreover, the integration locus of the Tn*7* transposon is well known [38] and does neither influence the growth characteristics of the labeled cell nor disrupt the function of another gene [39]. Labeling the biosensors in this way resulted in a significantly increased sensitivity of the fluorescence- relative to the turbidometric measurement. This enhancement could be even further improved by placing the *egfp* gene under the control of an even stronger promoter.

The general trend that emerged from analyzing the available data set was that both methods used to determine the biosensors' growth did not differ significantly in terms of their *accuracy* and *precision*. The two exceptions to this were the threonine biosensor that showed a greater precision for the turbidometric relative to the fluorescence measurement as well as the tryptophan biosensor, for which the fluorescence-based cell detection was slightly more accurate than the turbidometric OD measurement (Fig. 3b). While the ultimate cause for these discrepancies remain unclear, the proximate explanation for the latter observation was presumably the slightly lower coefficient of determination for the tryptophan OD measurement as compared to the corresponding fluorescence measurements (Table 2). Similar differences between OD- and fluorescence-based amino acid quantification have been observed previously for e.g. GFP-labeled lysine- [40] and methionine biosensors [2]. These findings highlight the need to carefully characterize the phenotype of a given biosensor with respect to the envisaged application, especially when absolute amino acid quantifications are required.

In conclusion, eleven amino acid biosensors that are based on single gene deletion mutants have been identified and phenotypically characterized. This array of publicly available biosensors fills a previous gap [18], [41] and can be applied to a broad range of different research contexts including e.g. the high-throughput screenings of mutant libraries [6], [14] or *in situ* measurements [11], [42], [43]. As such, these biosensors offer a simple and inexpensive way to quantitatively determine the bioavailable fraction of amino acids in very complex environments including soil, milk, blood, plant surfaces, and microbial biofilms [11]. While this study focused on demonstrating the general applicability of these biosensors, future work is necessary to further optimize the detection system e.g. by reducing the assay time or the detection limit. Moreover, it would be highly interesting to unravel whether mutants bearing multiple deletions can be generated that are specific auxotrophs for one of the remaining amino acids and/ or if a similar *in silico* analysis can also be successfully applied to design biosensors for other molecules of interest.

## Supporting Information

Figure S1 **Time-dependent growth of the eleven biosensors in the presence of the focal amino acid.** Biosensor growth was determined as culture turbidity (OD_600 nm_) in minimal medium supplemented with the focal amino acid (3 mM). Mean values (lines) of eight replicates (squares) are given.
(TIF)

Figure S2 **Development of GFP-fluorescence of the tryptophan biosensor cultivated in various concentrations of tryptophan.** Mean fluorescence emission of eight replicates is given as relative fluorescence units (RFU) after 12 h (triangles), 18 h (circles), and 24 h (squares) of growth.
(TIF)
