# Supplementary figures and images for: Design and Characterization of Auxotrophy-Based Amino Acid Biosensors
Source: PLoS One. 2012 Jul 19;7(7):e41349. doi: 10.1371/journal.pone.0041349 (PMC3400592; doi:10.1371/journal.pone.0041349)

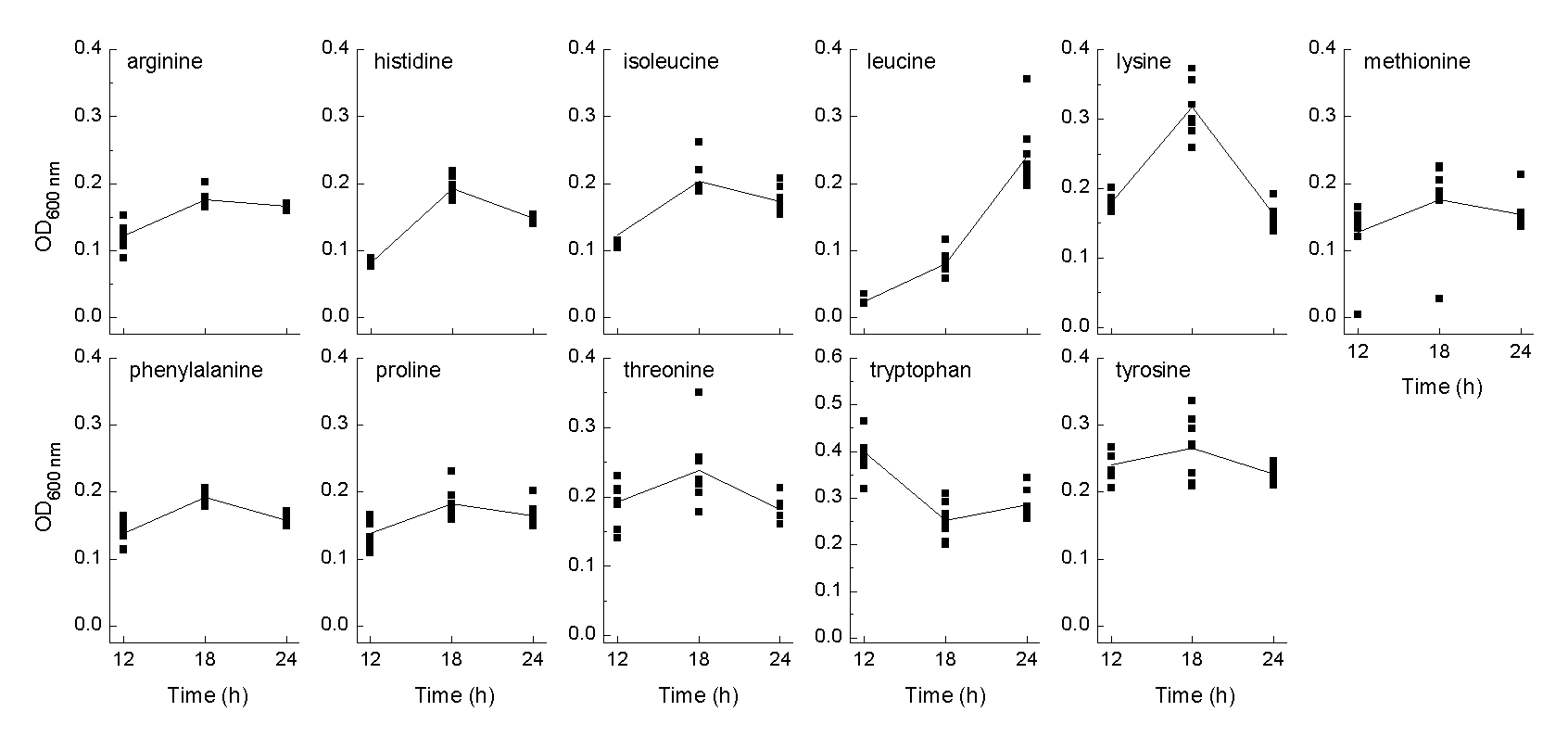

Supplement: Figure S1 — Time-dependent growth of the eleven biosensors in the presence of the focal amino acid. Biosensor growth was determined as culture turbidity (OD600 nm) in minimal medium supplemented with the focal amino acid (3 mM). Mean values (lines) of eight replicates (squares) are given. (TIF) [file pone.0041349.s001.tif]

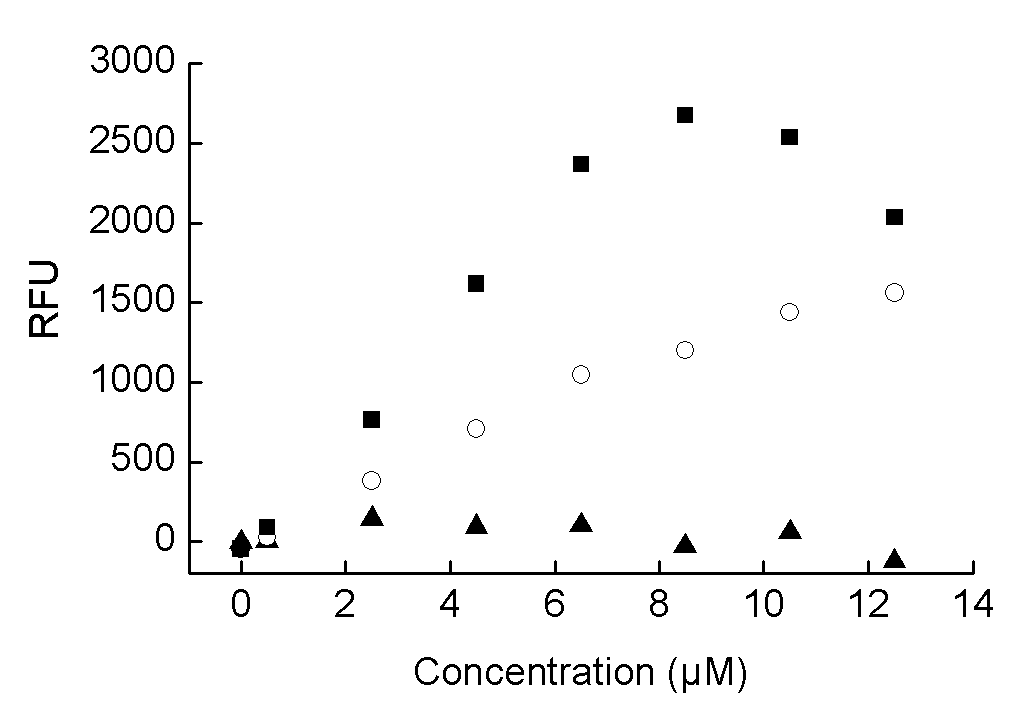

Supplement: Figure S2 — Development of GFP-fluorescence of the tryptophan biosensor cultivated in various concentrations of tryptophan. Mean fluorescence emission of eight replicates is given as relative fluorescence units (RFU) after 12 h (triangles), 18 h (circles), and 24 h (squares) of growth. (TIF) [file pone.0041349.s002.tif]
